# The lowest chromosome number in the family Pteromalidae (Hymenoptera: Chalcidoidea): the karyotype and other genetic features of Pachycrepoideus vindemmiae (Rondani, 1875)

**DOI:** 10.18699/vjgb-25-12

**Published:** 2025-02

**Authors:** V.E. Gokhman, A.S. Ryabinin, R.A. Bykov, Yu.Yu. Ilinsky

**Affiliations:** Russian Entomological Society, Moscow, Russia; Institute of Cytology and Genetics of the Siberian Branch of the Russian Academy of Sciences, Novosibirsk, Russia 3 Center for; Institute of Cytology and Genetics of the Siberian Branch of the Russian Academy of Sciences, Novosibirsk, Russia 3 Center for; Institute of Cytology and Genetics of the Siberian Branch of the Russian Academy of Sciences, Novosibirsk, Russia Center for Immunology and Cell Biology, Immanuel Kant Baltic Federal University, Kaliningrad, Russia

**Keywords:** Hymenoptera, Pteromalidae, parasitoids;, chromosomes, karyotype, DNA barcoding, endosymbionts, Hymenoptera, Pteromalidae, наездники, хромосомы, кариотип, ДНК-баркодинг, эндосимбионты

## Abstract

Various genetic features of the hitman strain of the widespread parasitoid of Drosophilidae (Diptera), Pachycrepoideus vindemmiae (Rondani, 1875) (Pteromalidae, Pachyneurinae) were studied. This strain was established and is maintained at the Institute of Cytology and Genetics of the Siberian Branch of the Russian Academy of Sciences (Novosibirsk, Russia). An analysis of air-dried chromosome preparations from prepupae of this parasitoid showed that it has n = 4 and 2n = 8 in males and females, respectively, which is the lowest known chromosome number in the family Pteromalidae. All chromosomes in the karyotype of this species are metacentric. The first and second chromosomes are of similar size, the remaining ones are substantially shorter. The same results were obtained for an additional strain of this species kept at the Moscow State University (Moscow, Russia). A comparison of the DNA sequence of the barcoding region of the mitochondrial cytochrome c oxidase (COI) gene of the hitman strain of P. vindemmiae with those available from the GenBank and BoLD databases demonstrated that this strain clustered together with conspecifics originating from China, Turkey and Italy. Despite certain endosymbionts being previously reported for the genus Pachycrepoideus Ashmead, 1904 as well as for P. vindemmiae itself, the hitman strain turned out to be free of endosymbiotic bacteria in the genera Arsenophonus Gherna et al., 1991, Cardinium Zchori-Fein et al., 2004, Rickettsia da Rocha-Lima, 1916, Spiroplasma Saglio et al., 1973 and Wolbachia Hertig, 1936. The above-mentioned results improve our knowledge of various genetic features of parasitoids of the family Pteromalidae and those of P. vindemmiae in particular.

## Abstract (ru)

Изучены различные генетические особенности культуры hitman широко распространенного паразитоида Drosophilidae (Diptera) Pachycrepoideus vindemmiae (Rondani, 1875) (Pteromalidae, Pachyneurinae), созданной и поддерживаемой в Институте цитологии и генетики Сибирского отделения Российской академии наук (Новосибирск, Россия). Анализ высушенных на воздухе хромосомных препаратов, полученных из предкуколок, показывает, что число хромосом для самцов и самок этого наездника составляет n = 4 и 2n = 8 соответственно; это наименьшее из известных для семейства Pteromalidae. Все хромосомы в кариотипе данного вида являются метацентриками. Первая и вторая хромосомы близки по размерам, остальные существенно короче. Такие же результаты получены еще для одной культуры этого вида, содержавшейся в Московском государственном университете (Россия). Сравнение последовательности ДНК баркодинг-участка, т. е. митохондриального гена цитохром c-оксидазы (COI) культуры hitman P. vindemmiae, с информацией, доступной в базах данных GenBank и BoLD, показало, что эта культура кластеризуется с конспецифичными особями, происходящими из Китая, Турции и Италии. Хотя некоторые эндосимбионты ранее указывались как для рода Pachycrepoideus Ashmead, 1904, так и для самого P. vindemmiae, оказалось, что культура hitman свободна от эндосимбиотических бактерий, принадлежащих к родам Arsenophonus Gherna et al., 1991, Cardinium Zchori-Fein et al., 2004, Rickettsia da Rocha-Lima, 1916, Spiroplasma Saglio et al., 1973 и Wolbachia Hertig, 1936. Вышеприведенные данные пополняют наши знания о различных генетических особенностях наездников семейства Pteromalidae, и в частности P. vindemmiae.

## Introduction

Parasitoid Hymenoptera are one of the most species-rich, taxonomically complicated and economically important groups of insects (Bebber et al., 2014; Forbes et al., 2018). In particular, the superfamily Chalcidoidea, with its exceptionally high morphological and ecological diversity, contains more than 27 thousand known species (Cruaud et al., 2024). Until recently, Pteromalidae represented the second largest family of Chalcidoidea with about four thousand members, but now it is subdivided into several smaller families (Huber, 2017; Burks et al., 2022). Nevertheless, karyotypes of less than twenty species of Pteromalidae s. l. have been studied up to now (Gokhman, 2024), as opposed to about 230 members of other Chalcidoidea (Gokhman, 2009, 2020). Among other Pteromalidae s. str. (hence Pteromalidae), we have recently studied the karyotype of a widespread parasitoid of Drosophilidae (Diptera), Pachycrepoideus vindemmiae (Rondani, 1875), using routine staining and morphometric analysis of chromosomes.

To ensure the precise identification of this species, which is of considerable interest as an effective agent of biological control (see, e. g., Bezerra Da Silva et al., 2019), we sequenced the barcoding region of the mitochondrial cytochrome c oxidase (COI ) gene of the same strain. In addition, many chalcids harbor maternally inherited bacterial endosymbionts that can cause various cytogenetic effects, for example, diploid thelytoky (Werren et al., 2008; Gokhman, Kuznetsova, 2018), which, in turn, can promote rapid fixation of chromosomal mutations. Specifically, these endosymbionts belong to the genera Arsenophonus Gherna et al., 1991, Cardinium Zchori-Fein et al., 2004, Rickettsia da Rocha-Lima, 1916, Spiroplasma Saglio et al., 1973 and Wolbachia Hertig, 1936 (Gavotte et al., 2007; Werren et al., 2008; Duron et al., 2010; Pilgrim et al., 2021; Nadal-Jimenez et al., 2023). Since the karyotype discovered in P. vindemmiae turned to be fairly aberrant for Pteromalidae (Gokhman, 2024) (see below), we have therefore conducted an additional study aimed at testing for the presence of various endosymbionts in the above-mentioned strain.

## Materials and methods

Origin of insects. The hitman strain of P. vindemmiae has been maintained in the Laboratory of Molecular Genetics of Insects (Institute of Cytology and Genetics of the Siberian Branch of the Russian Academy of Sciences, Novosibirsk, Russia) since 2018. It is reared on Drosophila melanogaster Meigen, 1830 (Diptera, Drosophilidae) under 19–22 °C and 60 ± 10 % humidity. The founder specimens of the strain were isolated by Dr. Sophia N. Panteleeva (Institute of Systematics and Ecology of Animals of the Siberian Branch of the Russian Academy of Sciences, Novosibirsk, Russia) from D. melanogaster pupae that were exposed at the Novosibirsk Arboretum in 2018. This strain can also be developed in the laboratory on Drosophila virilis Sturtevant, 1916 and D. mercatorum Patterson et Wheeler, 1942. For the karyotypic study, a few additional individuals were used from the laboratory stock kept at the Department of Evolutionary Theory (Moscow State University, Russia).

Karyotypic study. Chromosomal preparations were obtained from cerebral ganglia of seven male and four female parasitoid prepupae generally following the protocol developed by Imai et al. (1988) with certain modifications. Ganglia were extracted from insects dissected in 0.5 % hypotonic sodium citrate solution containing 0.005 % colchicine. The extracted ganglia were then transferred to a fresh portion of hypotonic solution and incubated for 30 min at room temperature. The material was transferred onto a pre-cleaned microscope slide using a Pasteur pipette and then gently flushed with Fixative I (glacial acetic acid : absolute ethanol : distilled water 3:3:4). The tissues were disrupted using dissecting needles in an additional drop of Fixative I. A drop of Fixative II (glacial acetic acid : absolute ethanol 1:1) was applied to the center of the area, and the more aqueous phase was blotted off the edges of the slide. The same procedure was performed with Fixative III (glacial acetic acid). The slides were then dried for approximately half an hour and stored at room temperature. Chromosome preparations were stained overnight with freshly prepared 3 % Giemsa solution.

Metaphase plates were analyzed under a Zeiss Axioskop 40 FL epifluorescence microscope (Carl Zeiss, Germany). Images of chromosomes from 21 haploid and 31 diploid mitotic divisions were taken with Zeiss AxioCam 208 digital camera using ZEN software version 3.0. To prepare illustrations, the resulting images were arranged and enhanced with GIMP 2.10. KaryoType software version 2.0 was also used for taking chromosome measurements from five diploid metaphase plates of good quality. The chromosomes were classified following the guidelines provided by Levan et al. (1964).

Molecular study. For barcoding and screening for endosymbionts, DNA was extracted from at least five pooled specimens. During 2018–2024, the hitman strain was tested four times for maternally inherited endosymbionts in conjunction with replacements of the host strains

Parasitoid specimens were homogenized in 200 μl extraction buffer (0.1M NaCl, 10 mM Tris HCl (pH 8.0), 25 mM EDTA, 0.5 % SDS) and incubated at 56 °C for an hour. DNA was then salted out with 100 μl 5M potassium acetate/3M acetic acid with further precipitation and dissolution in 100 μl double-distilled water. All PCRs were carried out in 20 μl mix containing chemicals from the Biomaster HS-Taq PCR kit (Biolabmix, Russia), together with a specific primer set and genomic DNA, with the following cycling conditions: an initial denaturation at 95 °C for 5 min, 35 cycles at 95 °C – 15 sec, 53 °C – 1 min for COI and Spiroplasma, 55 °C – 30 sec for Cardinium and 40 sec for Arsenophonus and nested PCRs followed by elongation at 72 °C – 40 sec, and a final elongation at 72 °C for 2 min. The presence of Rickettsia and Wolbachia was checked by nested PCR, with 15 cycles for the first round and 25 cycles for the second round; 1 μl of the reaction volume from the first round was used in the second one. Primers used in this study are listed in Table 1. The amplicon was purified with exonuclease (ExoI) (New England Biolabs, USA), and sequenced using the BrilliantDye ™ Terminator Cycle Sequencing Kit (NimaGen, The Netherlands). The sequence of the COI gene was deposited in GenBank under accession number PP727399.

**Table 1.**
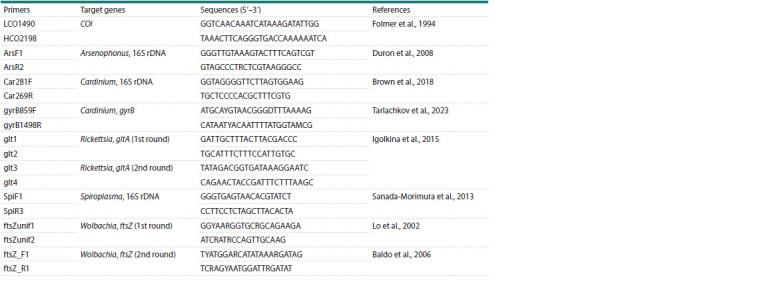
Primers used in this study

We retrieved all sequences of the barcoding fragment of the COI gene deposited under the name of P. vindemmiae in the Barcode of Life Database (BoLD) (Ratnasingham, Hebert, 2007) and GenBank. Using BLAST nucleotide search (https:// blast.ncbi.nlm.nih.gov), we also found COI gene sequences for a few other species with the highest similarity with those of P. vindemmiae. These sequences were also included into the analysis. The maximum likelihood (ML) phylogenetic tree of the COI gene was reconstructed using MEGA6 software (Tamura et al., 2013) under the General Time Reversible model as the best fit and bootstrapping at 1,000 iterations.

## Results

**Fig. 1.**
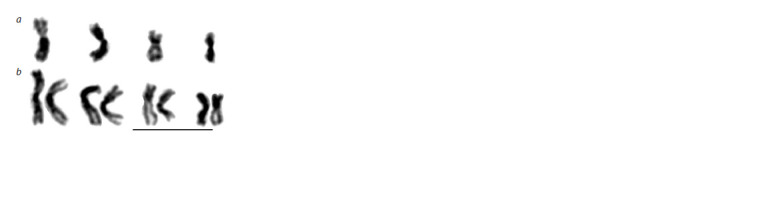
Karyograms of P. vindemmiae: a – male (haploid), b – female (diploid). Bar = 10 μm.

The haploid karyotype of P. vindemmiae harbors four metacentric chromosomes (n = 4), although the first chromosome is close to a submetacentric one (Fig. 1a, Table 2). The second metacentric chromosome is similar in length to the first one, the remaining chromosomes are distinctly shorter. Consequently, the diploid karyotype of this species contains eight chromosomes (2n = 8) (Fig. 1b). No obvious chromosomal difference was detected between the strains from Novosibirsk and Moscow 

**Table 2.**
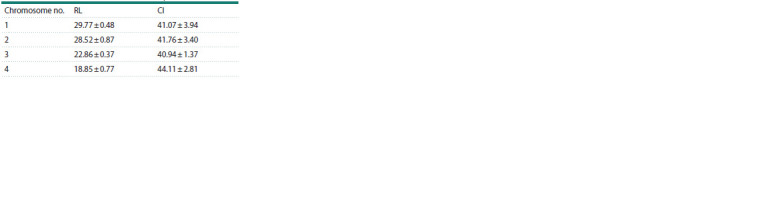
Relative lengths (RLs) and centromeric indices (CIs) of P. vindemmiae chromosomes (mean ± SD)

**Fig. 2.**
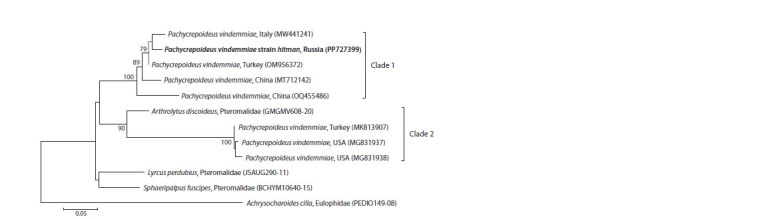
The maximum likelihood (ML) phylogenetic tree of Pachycrepoideus COI mitochondrial DNA sequences (577 bp region) reconstructed with the GTR+G model GenBank and/or BoLD accession numbers as well as origins of samples are indicated. The hitman strain indicated in bold. COI sequences of Arthrolytus discoideus (Nees, 1834) (Pteromalidae), Achrysocharoides cilla (Walker, 1839) (Eulophidae), as well as Lyrcus perdubius (Girault, 1916) and Sphaeripalpus fuscipes (Walker, 1833) (Pteromalidae) were used as outgroups. Bootstrap values higher than 75 (1,000 iterations) are indicated. The scale bar denotes the number of substitutions per site.

We sequenced 652 bp of the mitochondrial COI gene of the hitman strain and reconstructed the ML phylogenetic tree, which included all annotated sequences available for P. vindemmiae (Fig. 2). There are two clades on the ML phylogenetic tree, in which the hitman strain is clustered with conspecifics from China, Turkey and Italy (Clade 1), while another cluster is formed by two strains from the USA as well as by another one from Turkey (Clade 2). However, the latter clade also turned out to include Arthrolytus discoideus (Nees, 1834) (Pteromalidae, Pteromalinae). We did not find any molecular evidence for the presence of any checked endosymbiont, i. e., Arsenophonus, Cardinium, Rickettsia, Spiroplasma and Wolbachia.

## Discussion

Among other members of Pteromalidae s. l., n = 4 was reported only for Spalangia endius Walker, 1839 (Spalangiidae) from Thailand (Kitthawee, Vasinpiyamongkol, 2002). However, the same chromosome number found in P. vindemmiae represents the lowest n value known for the family Pteromalidae (Gokhman, 2024), with other members of the family having n = 5–7. The most frequent chromosome number, which is characteristic of most species of Pteromalidae, is n = 5 (Gokhman, 2024). Although it is unclear at present which n value can be considered ancestral for the family, this is almost certainly not n = 4, i. e., the chromosome number found in P. vindemmiae. Moreover, the latter species and all other Pteromalidae with known karyotypes belong to the subfamilies Pachyneurinae and Pteromalinae respectively (Burks et al., 2022). It is therefore not surprising that P. vindemmiae demonstrates deviating chromosomal characters. Moreover, strong behavioral and molecular differences between this species and many other Pteromalidae were already noted by previous authors (van den Assem, 1974; Huang et al., 2023).

Taking into account the large genetic distance between Clades 1 and 2, we suggest that the latter clade does not actually belong to P. vindemmiae. Indeed, according to the available data, studied samples of P. vindemmiae that belong to the second clade appear to be more closely related to Arthrolytus discoideus than to the strains of Clade 1 of P. vindemmiae (Fig. 2). However, the pteromalid genera Arthrolytus Thomson, 1878 and Pachycrepoideus Ashmead, 1904 belong to different subfamilies (see above), and therefore identifications of these samples of P. vindemmiae may well be wrong.

Maternally inherited endosymbionts are constantly transferred to the offspring, and therefore they can be effectively considered facultative components of the host genome. Currently, Arsenophonus remains the only endosymbiont genus detected in P. vindemmiae (Duron et al., 2010; Nadal-Jimenez et al., 2023). Moreover, Wolbachia and Rickettsia were also previously reported for the genus Pachycrepoideus (Gavotte et al., 2007; Pilgrim et al., 2021). However, the hitman strain appears to be free from all these endosymbionts. Since we have recurrently checked this strain for any endosymbiotic microorganisms for a few years starting from 2018 (see above), we can assume that the hitman strain was neither initially infected with these endosymbionts nor obtained them via different hosts in the process of rearing.

## Conclusion

Chromosome preparations from prepupae of Pachycrepoideus vindemmiae showed that it has n = 4 and 2n = 8 in males and females, respectively, which are the lowest known chromosome numbers in the family Pteromalidae. A comparison of the DNA sequence of the COI barcoding region of the hitman strain of P. vindemmiae demonstrated that this strain clustered together with conspecifics originating from China, Turkey and Italy. The hitman strain turned out to be free of endosymbiotic bacteria in the genera Arsenophonus, Cardinium, Rickettsia, Spiroplasma and Wolbachia. These results improve our knowledge of various genetic features of parasitoids of the family Pteromalidae and those of P. vindemmiae in particular.

## Conflict of interest

The authors declare no conflict of interest.
